# Synergistic Disruption of Foodborne Pathogen Biofilms by Oregano Essential Oil and Bacteriophage phiLLS: Atomic Force Microscopy Insights

**DOI:** 10.3390/molecules30173552

**Published:** 2025-08-30

**Authors:** Ana Karina Kao Godínez, Carlos Regalado-González, Claudia Villicaña, José Basilio Heredia, José Benigno Valdez-Torres, María Muy-Rangel, Monserrat Escamilla-García, Josefina León-Félix

**Affiliations:** 1 Centro de Investigación en Alimentación y Desarrollo, A.C., Culiacán 80110, Mexico; akao221@estudiantes.ciad.mx (A.K.K.G.); jbheredia@ciad.mx (J.B.H.); jvaldez@ciad.mx (J.B.V.-T.); mdmuy@ciad.mx (M.M.-R.); 2 Facultad de Química, Universidad Autónoma de Querétaro, Cerro de las Campanas, Querétaro 76010, Mexico; regcarlos@gmail.com (C.R.-G.); monserrat.escamilla@uaq.mx (M.E.-G.); 3 SECIHTI-Centro de Investigación en Alimentación y Desarrollo, A.C., Culiacán 80110, Mexico; maria.villicana@ciad.mx

**Keywords:** atomic force microscopy, bacteriophage phiLLS, foodborne pathogens, oregano essential oil, biofilm disruption and synergy

## Abstract

Foodborne pathogenic biofilms pose significant challenges to food safety due to their enhanced resistance to conventional antimicrobial agents. In this study, we evaluated the synergistic antibiofilm activity of oregano essential oil (OEO) from *Lippia graveolens* and the lytic bacteriophage phiLLS against six foodborne bacteria. GC–MS analysis achieved a 100% identification ratio, revealing that OEO was mainly composed of carvacrol (58.9%), p-cymene (28.6%), γ-terpinene (2.9%), and caryophyllene (2.6%). The MIC and MBC of OEO were 1 and 2 mg/mL, respectively, for all strains except *E. coli* BALL1119 (MIC = 2 mg/mL). We assessed biofilm biomass by crystal violet (CV) staining and metabolic activity using the TTC assay under both individual and combined treatments, monitored 9-hour planktonic growth kinetics to calculate Bliss and HSA synergy indexes, and employed atomic force microscopy (AFM) to visualize nanoscale alterations in *Staphylococcus aureus* and *Escherichia coli* BALL1119 biofilms. Combined OEO (2 mg/mL) and phiLLS (MOI 1) treatments achieved significantly greater biofilm biomass reduction than single agents, notably yielding >70% inhibition of *S. aureus* biofilms (*p* < 0.05) and a Bliss synergy index of 10.8% in *E. coli* BALL1119 growth kinetics, whereas other strains were additive. In biofilm assays, *S. aureus* and *Salmonella* spp. showed the highest reductions in biomass (CV) (71.0% and 67.8%, ΔHSA = 27.0% and 17.4%; ΔBliss = 21.1% and 13.8%) and metabolic activity (TTC) (68.6% and 48.5%). AFM revealed that OEO alone smoothed the extracellular matrix (averaging a 35% reduction in roughness), whereas the combined treatment caused fracturing (≈68 nm roughness) and prominent lytic pits. Although variability in *S. aureus* biofilm architecture precluded statistically significant pairwise comparisons, AFM topography and consistent trends in Ra/Rz parameters provided clear visual corroboration of the significant reductions detected by CV and TTC assays. These complementary data indicate that OEO primes the biofilm matrix for enhanced phage-mediated collapse, offering a green, two-step strategy for controlling resilient foodborne biofilms.

## 1. Introduction

Bacterial biofilms are three-dimensional communities of cells encased in a self-produced extracellular polymeric substance (EPS) matrix that confers enhanced resistance to antimicrobials and environmental stresses compared to planktonic cells [1,2]. In food processing environments, biofilm-forming pathogens such as *Escherichia coli*, *Salmonella* spp., *Listeria monocytogenes*, and *Staphylococcus aureus* pose a significant threat to public health, causing persistent contamination of equipment and food surfaces [3,4]. Foodborne diseases remain a persistent global public health challenge, affecting millions of people annually and causing significant economic losses [5,6,7]. According to the World Health Organization, nearly 1 in 10 people worldwide fall ill each year after consuming contaminated food, resulting in over 420,000 deaths, including approximately 125,000 deaths of children under five years old [8,9]. Fresh produce, in particular, has been identified as a major vehicle for pathogenic bacteria such as *E. coli* O157:H7, *S. enterica*, *L. monocytogenes*, *S. aureus*, and *B. cereus* [10,11]. Between 2010 and 2019, a marked increase in multistate foodborne outbreaks linked to fresh produce was reported in the United States, often associated with imported produce from Mexico and Turkey [12,13,14].

The alarming rise of antimicrobial resistance represents one of the main challenges in controlling foodborne pathogens, exacerbated by the extensive use of antibiotics in food production [9,15]. Without effective interventions, resistant infections are projected to cause up to 10 million deaths annually by 2050 [16]. The ability of certain pathogens to form biofilms on fresh produce and abiotic surfaces further aggravates this problem, conferring resistance up to 1000-fold higher than that of planktonic cells [17,18,19]. These complex structures hinder eradication and promote the persistence of contamination in food processing environments.

Plant-derived essential oils (EOs) have emerged as appealing “green” antimicrobials due to their broad-spectrum activity and low toxicity. Oregano essential oil (OEO) from *Lippia graveolens*, rich in phenolic monoterpenes such as carvacrol and thymol, disrupts cell membranes and interferes with metabolic processes, significantly reducing biofilm biomass at subinhibitory concentrations [20,21]. However, the hydrophobic nature of EOs and the diffusional barrier imposed by the EPS matrix often limit their penetration into established biofilms [3,22].

Bacteriophage therapy offers high specificity and the capacity to replicate within host cells, yet standalone phage treatments can yield variable biofilm reductions depending on biofilm age, structure, and phage attributes [23,24]. PhiLLS, a lytic *Siphoviridae* phage isolated from wastewater in Culiacán, México, demonstrates a broad host range (~68% of 57 tested *E. coli* strains), a burst size of ~176 PFU/cells, a latent period of 15 min, and a ≈107 kb genome lacking known virulence or toxin genes, supporting its safety as a biocontrol agent [25]. Phage-derived depolymerases can degrade specific components of the biofilm matrix, enhancing access to host bacteria [26]. However, these enzymes alone are often insufficient for completely eradicating biofilms [27]. Engineered phages expressing quorum-quenching enzymes can inhibit biofilm formation in mixed-species communities [28]. Interestingly, even disintegrated phages without lytic activity can degrade biofilms, likely due to the enzymatic activities of their structural proteins [14]. Genomic analysis has revealed genes encoding various enzymes that may contribute to biofilm degradation [29]. Combined therapies using phages or phage-derived products with other antimicrobial agents show potential as practical approaches for biofilm removal and treatment of associated infections [26,27].

Recent studies suggest that combining EOs with bacteriophages can produce synergistic antibiofilm effects (fractional inhibitory concentration index ΣFIC ≤ 0.5), achieving greater reductions in biofilm biomass than individual treatments [30]. These studies have highlighted that combining essential oils with bacteriophages not only limits bacterial growth but also impairs biofilm formation [24,30,31]. These combined treatments have been shown to modulate the expression of virulence, adhesion, and quorum-sensing genes in pathogenic bacteria, thereby reducing their infectious potential. Biofilm eradication in these investigations is typically quantified using microtiter-plate assays. Treated biofilms are stained with 0.1% crystal violet, washed, and de-stained with ethanol, and absorbance read is at 595 nm to determine residual biomass [32]. Complementary methods, such as TTC metabolic assays or CFU enumeration, help distinguish between matrix dispersal and true cell death. In this context, the Mexican oregano (*L. graveolens*) essential oil, rich in carvacrol and thymol [33], and the lytic bacteriophage phiLLS [25], emerge as promising candidates for a dual “green” approach to biofilm control [34,35].

Atomic force microscopy (AFM) provides nanoscale insights into biofilm surface topography and mechanical properties. AFM analyses of *S. aureus* and *Pseudomonas aeruginosa* biofilms have revealed treatment-induced changes in matrix roughness and cell morphology, elucidating the physical impact of antimicrobials on EPS integrity [36,37].

Here, we investigate the synergistic antibiofilm activity of OEO and bacteriophage phiLLS against biofilms formed by six foodborne pathogens. It was to (i) determine the minimum inhibitory and bactericidal concentrations of OEO, (ii) quantify biofilm inhibition and eradication under solo and combined treatments using the Haney et al. microtiter assay [38], (iii) monitor planktonic growth kinetics over 9 h, and (iv) employ AFM to visualize nanoscale alterations in the biofilm architecture of *S. aureus* and *E. coli*. Our findings aim to elucidate the mechanisms driving synergy and inform the development of eco-friendly biofilm control strategies in food safety.

## 2. Results

### 2.1. Composition of the Essential Oil

Thirteen constituents, representing 100.0% of the total oil, were identified (Table 1). The major components were carvacrol (58.90%), p-cymene (28.62%), γ-terpinene (2.89%), and caryophyllene (2.59%), while the remaining compounds were present at <1%.

### 2.2. Phage Sensitivity to OEO

The stability of phage phiLLS in oregano essential oil was assessed by incubating the phage with varying concentrations of OEO (4 mg/mL, 2 mg/mL, 1 mg/mL, 0.5 mg/mL, and ethanol at 2%). At 24 h post-incubation, there was no significant effect of OEO at concentrations of 0.5–4 mg on phage phiLLS viability.

**Figure 1:**
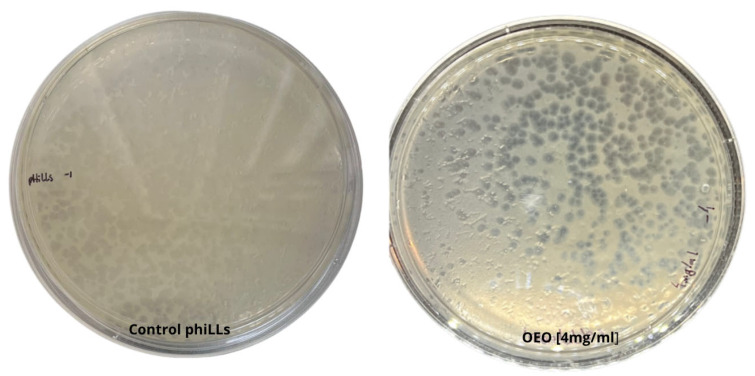
Viability of bacteriophage phiLLS after incubation with 4 mg/mL oregano essential oil (OEO) in a soft-agar overlay assay (10^−4^ dilution). The presence of numerous, well-formed plaques (titer 1.8 × 10^9^ PFU/mL) indicates full retention of infectivity, with no reduction in plaque count or morphology compared to OEO-free controls.

A 10^−4^ dilution of phiLLS was mixed with 4 mg/mL oregano essential oil (OEO) in molten soft agar and overlaid onto a bacterial lawn. The presence of numerous, well-formed plaques across the plate (titer 1.8 × 10^9^ PFU/mL) of phiLLS demonstrates that it remains fully viable at this OEO concentration and dilution, with no observable reduction in plaque count or morphology relative to OEO-free controls (Figure 1).

### 2.3. MIC and MBC Values of OEO

The antimicrobial activity of oregano essential oil (OEO) was determined against *E. coli* O157:H7, *E. coli* BALL 1119, *Salmonella* spp., *L. monocytogenes*, *S. aureus*, and *B. cereus*. OEO was tested at concentrations ranging from 4.0 to 0.025 mg/mL using the broth microdilution method. MIC values were 1 mg/mL for all strains except *E. coli* BALL 1119, which exhibited an MIC of 2 mg/mL. MBC values were consistently 2 mg/mL for all strains (Table 2). These results guided the selection of 2 mg/mL OEO for subsequent global assays, corresponding to the MBC for all strains and the MIC for *E. coli* BALL 1119. Untreated cultures served as negative controls, and medium-only wells served as sterility controls.

### 2.4. Combined Application of OEO and Phage

#### 2.4.1. Growth Kinetics: Combined vs. Individual Treatments

**Figure 2:**
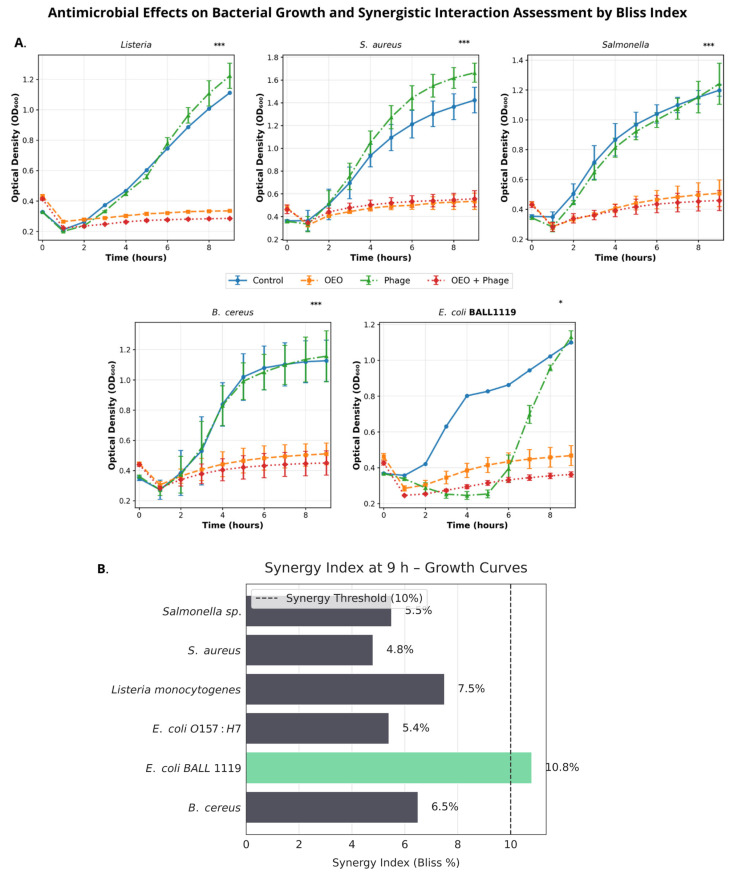
Antimicrobial effects on bacterial growth kinetics and Bliss synergy analysis. (**A**) Growth kinetics of foodborne pathogens under different treatments: control (blue), oregano essential oil (OEO, orange), bacteriophage phiLLS (green), and OEO + phiLLS (red). Curves represent mean ± SD (n = 3). Asterisks indicate statistically significant differences compared to the untreated control (* *p* < 0.05, *** *p* < 0.001; one-way ANOVA with Tukey’s HSD), assessed at 9 h. (**B**) Bliss synergy index (%) calculated from the growth kinetics data. A dashed vertical line marks the 10% synergy threshold. Dark-grey bars denote additive interactions (≤10%), and the green bar indicates the only synergistic case observed in growth kinetics (*E. coli* BALL 1119, 10.8%). Exact Bliss values are labeled at the end of each bar.

The growth kinetics of six foodborne pathogens under individual (OEO [2 mg/mL], phiLLS MOI 1) and combined (OEO + phiLLS) treatments were evaluated by measuring the optical density at 600 nm (OD_600_) over 9 h. We first compared the growth curves and derived key kinetic parameters, including specific growth rate (μ) and maximum density (OD_max_), to assess treatment efficacy. Subsequent statistical analyses (one-way ANOVA and Tukey’s HSD) identified significant differences in μ among treatments for each pathogen. Kinetic slope analysis for *E. coli* BALL 1119 (Figure 2A) shows that between 1 and 4 h, the phage treatment drives rapid initial inhibition (slope = 18.7% h^−1^), but its efficacy declines from 4 to 9 h (negative slope). In contrast, OEO and the combined OEO + phage treatment maintain gently positive slopes throughout the entire 1–9 h window.

**Figure 3:**
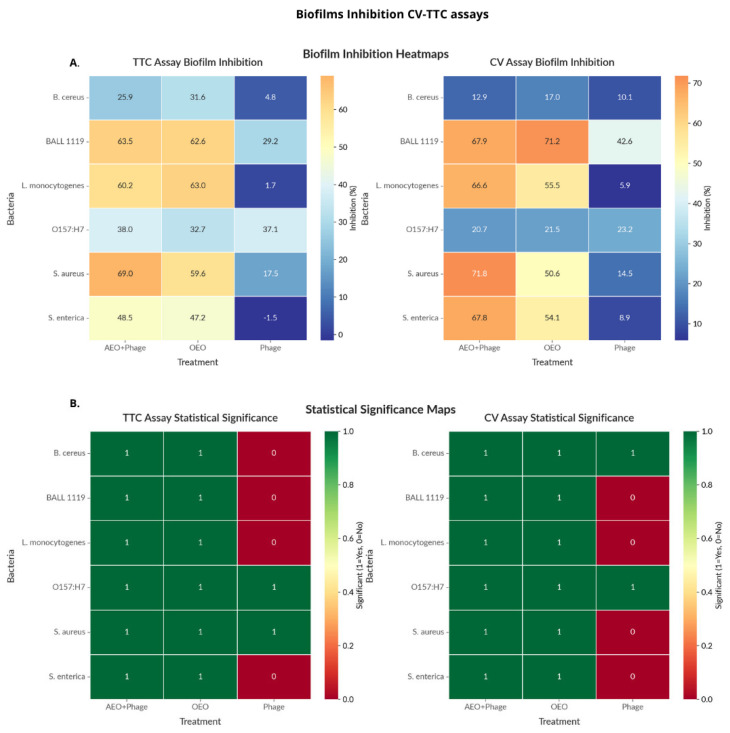
Biofilm assays: biomass (CV) and metabolic activity (TTC). Heatmaps showing percent reduction in biomass (CV) and metabolic activity (TTC) across all bacteria–treatment combinations (**A**). The significance maps (**B**) show which treatments produced statistically significant inhibition (green = significant, red = not significant) for each bacteria–treatment combination.

**Figure 4:**
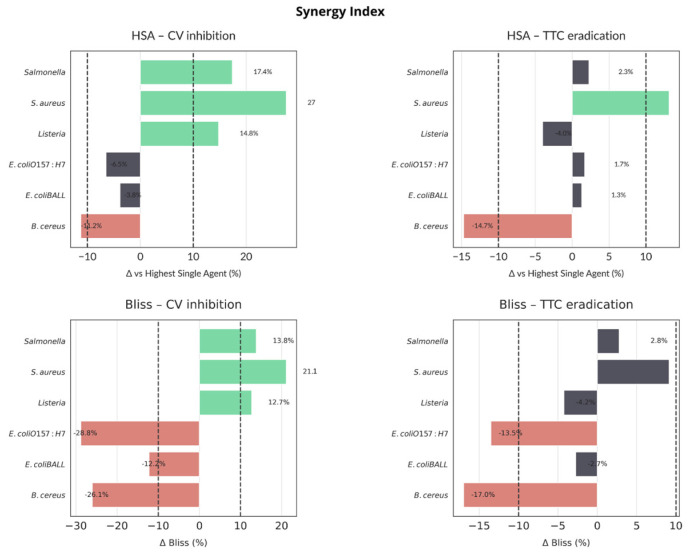
Synergy Assessment of OEO + phage. The Highest-Single-Agent (HSA) and Bliss independence models were applied to the OEO + phage phiLLS combination across six food-borne pathogens. *ΔBliss and ΔHSA* panels: grey = additive (|Δ| ≤ 10%), green = synergistic (Δ > 10%), red = antagonistic (Δ < –10%). Dashed lines at ±10% denote thresholds.

The Bliss synergy index (%) at 9 h for the combined OEO + phiLLS treatment is shown in Figure 2B; a dashed vertical line indicates the 10% synergy threshold. In the context of growth kinetics only, dark-grey bars denote additive interactions (*S. aureus*, *Salmonella* spp., *L. monocytogenes*, *E. coli* O157:H7, and *B. cereus*, ≤ 10%), and the green bar indicates the sole synergistic case (*E. coli* BALL 1119, 10.8%). Synergy observed here does not necessarily reflect results from biofilm assays (Figure 3 and Figure 4), which are described separately below.

#### 2.4.2. Biofilm Assays

The effect of phage phiLLS in combination with OEO on preformed biofilms was quantified using biomass (CV) and metabolic activity (TTC) measurements and analyzed under the Highest Single Agent (HSA) [39] and Bliss independence models [40]. Across the six foodborne pathogens (Figure 3), the OEO + phiLLS combination significantly reduced biomass in *S. aureus* and *Salmonella* spp. (*p* < 0.05, Tukey’s HSD), achieving maximum biomass reductions of 71.0% and 67.8%, respectively, and corresponding metabolic activity reductions of 68.6% and 48.5%. Average inhibition across both endpoints was ~48% for *S. aureus* and ~44% for *Salmonella* spp.

In contrast, *B. cereus* biofilms were largely refractory, with no treatment exceeding a 50% reduction in either endpoint, and no significant differences from the control in TTC were noted. *E. coli* strains showed moderate biomass reduction (≤63%) but negligible metabolic inactivation. Overall, *S. aureus* and *Salmonella* emerged as the most susceptible species, with biomass reduction generally exceeding metabolic inactivation.

Synergy analysis (Figure 4) indicated convergent synergy (Δ > 10% in both HSA and Bliss) for *L. monocytogenes*, *S. aureus*, and *Salmonella* spp. in biomass reduction (CV). Only *S. aureus* exhibited synergy in metabolic inactivation (TTC). *B. cereus* showed consistent antagonism (Δ < –10%) across both models and endpoints, while *E. coli* strains fell into the additive range. Applying both HSA and Bliss models allowed complementary perspectives. HSA benchmarks the best single agent, while Bliss accounts for expected additive effects and more stringently identifies antagonism when one agent is weak.

Synergy analysis of biofilm disruption by the OEO + phiLLS combination revealed consistent synergistic effects (Δ > 10%) against *L. monocytogenes*, *S. aureus*, and *Salmonella* spp. in the CV biomass reduction assay (both HSA and Bliss models) (Figure 4), with *S. aureus* also exhibiting synergy in the TTC inhibition metabolic activity assay. In contrast, *Bacillus cereus* displayed clear antagonism (Δ < –10%) under both HSA and Bliss criteria in both assays, and both *E. coli* strains fell into the additive range. Bliss penalizes low phage activity more severely than HSA (Figure 4). Overall, synergy was relatively rare (only three of six species in CV, one in TTC), while antagonistic interactions predominated for species poorly inhibited by phage alone. These results underscore the importance of applying both HSA and Bliss models. HSA offers an intuitive comparison to the best single agent, whereas Bliss corrects for expected additive effects and more stringently flags antagonism when one agent is weak. Convergent synergy calls (e.g., for *S. aureus* and *Salmonella*) provide high confidence in combination efficacy, whereas divergent classifications warrant further kinetic investigation or concentration optimization.

The treatments showed significantly high (77.8%) biofilm inhibition in both assays and limited synergy. Only three out of six bacteria showed synergistic effects (*S. aureus*, *Listeria,* and *Salmonella* in the CV assay). Antagonistic effects were particularly noted for *B. cereus*. Regarding treatment effectiveness, the combination treatment (OEO + Phage) generally performed well but did not always exceed expected additive effects.

### 2.5. Evaluation of the Efficacy of the Combined phiLLS and OEO Under AFM for E. coli 1119 and S. aureus Biofilms

*E. coli* BALL1119 was selected as a representative susceptible host of phiLLS, while *S. aureus* was chosen to investigate the intriguing antibiofilm effects observed in a non-host species. The surface architecture of *E. coli* BALL 1119 and *S. aureus* biofilms was examined following treatment with phage phiLLS (MOI 1), oregano essential oil (OEO, 2 mg/mL), and their combination using atomic force microscopy (AFM) in tapping mode. Untreated biofilms served as the control for each species.

**Figure 5:**
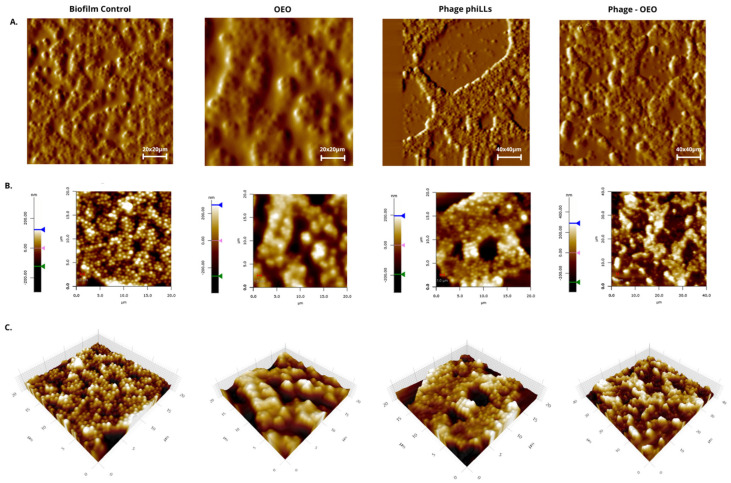
Representative AFM topographical analysis of *S. aureus* biofilms under different treatments. Biofilms were imaged in tapping mode under four conditions: untreated control, oregano essential oil (OEO, 2 mg/mL), bacteriophage phiLLS, and combined phiLLS + OEO. (**A**) Topographic error signal images (scan sizes: 20 µm × 20 µm and 40 µm × 40 µm) displaying overall surface texture and microcolony arrangement for each treatment. (**B**) Corresponding height maps (20 µm × 20 µm) with a continuous color gradient (−200 to +200 nm), and color bars indicating minimum (black/green arrow), midpoint (brown/magenta arrow), and maximum (white/blue arrow) elevations; lateral scale bars = 1 µm. (**C**) Three-dimensional surface renderings of the same 20 µm × 20 µm areas, illustrating treatment-dependent changes in biofilm morphology. Control biofilms exhibit dense mounds and channels; OEO flattens and smooths the surface; phiLLS creates localized pits and erosive features; phiLLS + OEO produces pronounced valleys and sharp peaks, reflecting synergistic disruption of the extracellular matrix. Color gradients indicate surface elevation (nm), with white tones corresponding to the highest points and black to the deepest.

**Figure 6:**
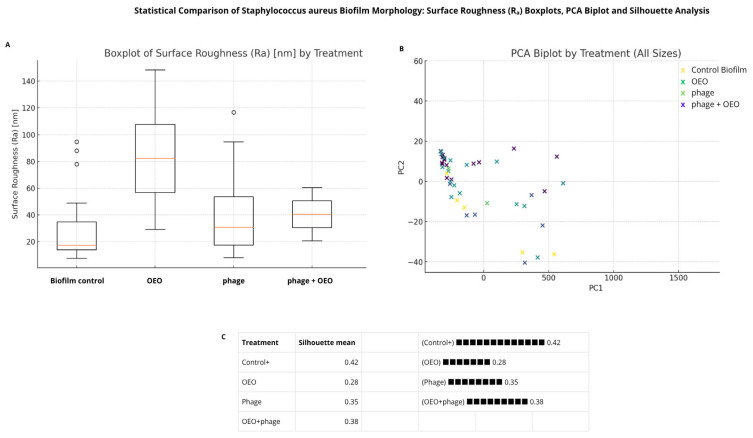
Statistical analysis of *S. aureus* biofilm morphology under four treatments (Control+, OEO, phiLLS, phiLLS + OEO) using AFM-derived surface parameters. (**A**) Boxplots of surface roughness (R_a_) by treatment: OEO and phiLLS + OEO tend to increase median R_a_ relative to Control+, while phiLLS alone shows reduced R_a_; not pairwise differences remained significant after Bonferroni correction. (**B**) PCA biplot of all samples (PC1 vs. PC2), with points colored by treatment. PC1 (driven primarily by Rz and R_a_) separates untreated controls (low PC1, high PC2) from phage and combination treatments (positive PC1), indicating greater surface disruption. (**C**) Mean Silhouette scores for each treatment group, quantifying clustering cohesion and separation: Control+ exhibits the highest cohesion, OEO the lowest, and phiLLS + OEO intermediate. Together, these multivariate analyses indicate that phiLLS + OEO produces the most consistent disruption of the EPS matrix, followed by phiLLS alone, whereas untreated biofilms remain tightly clustered.

**Figure 7:**
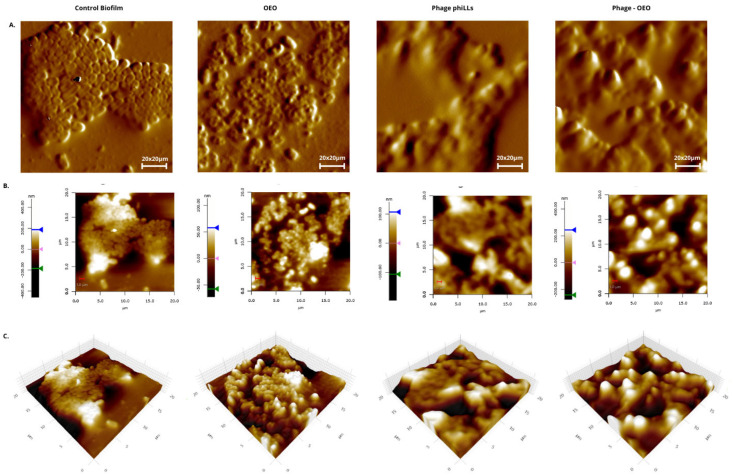
AFM topographical comparison of *Escherichia coli* BALL1119 biofilms under different treatments. (**A**) Two-dimensional forward scan height images (20 µm × 20 µm) for Control (untreated), OEO (oregano essential oil), phiLLS (bacteriophage phiLLS), and combined OEO + phiLLS. Each panel displays cell microcolonies and EPS distribution in false-color height. (**B**) Corresponding 2D height maps with color scales (nm) indicating z-range; lateral axes in µm and 1 µm scale bar. Arrows on the scale bars mark the minimum (green), midpoint (magenta), and maximum (blue) heights. (**C**) Three-dimensional reconstructions of the same 20 µm × 20 µm areas, illustrating surface morphology and roughness differences. Control exhibits mature “mounds” and channels; OEO shows a flattened and rougher surface; phiLLS displays localized erosion and pits; OEO + phiLLS reveals deep valleys and sharp peaks. Treatment abbreviations: OEO, oregano essential oil; phiLLS, bacteriophage phiLLS.

Figure 5, Figure 6 and Figure 7 show that the topographic and 3D renderings reveal that untreated biofilms from both species retained a compact, mound-like structure with dense and continuous extracellular matrix, indicating an intact and undisturbed surface. In contrast, phiLLS-treated biofilms exhibited localized erosive features and pits, while OEO treatment caused general surface flattening and matrix loosening. Notably, the combined treatment resulted in pronounced morphological changes, with deep valleys, fragmented ridges, and overall disruption of the matrix architecture, particularly evident in *S. aureus*.

Quantitative analysis of AFM-derived parameters, including surface roughness (Ra) and maximum peak height (Rz), was performed for both species. For *E. coli* BALL 1119, one-way ANOVA followed by Tukey’s HSD showed statistically significant increases in Ra for the combined treatment compared to both individual treatments and the untreated control (*p* < 0.001). In *S. aureus*, ANOVA assumptions were not met; therefore, non-parametric tests were applied. Although these did not yield statistically significant pairwise differences after Bonferroni correction, consistent trends pointed to greater surface heterogeneity in the OEO and phiLLS + OEO groups.

Principal component analysis (PCA, Figure 6) further supported these patterns, clearly separating untreated biofilms from treated ones along PC1, which was associated with increased matrix disruption. Silhouette scores indicated higher structural cohesion in untreated biofilms and reduced clustering in the OEO group. Collectively, these results hypothesize that while *S. aureus* is not a direct host of phiLLS, the combination with OEO amplifies matrix disintegration, likely through synergistic interactions involving EPS destabilization and enhanced access of phage-associated lytic enzymes.

Figure 7 shows that untreated control biofilms exhibited a well-organized architecture, with dense microcolonial mounds and interconnected EPS channels, hallmarks of a mature and cohesive matrix. Treatment with OEO alone produced a flatter yet rougher surface, consistent with partial destabilization of the biofilm structure. In contrast, phiLLS treatment alone caused localized pitting and surface erosion, characteristic of lytic phage activity in susceptible hosts.

**Figure 8:**
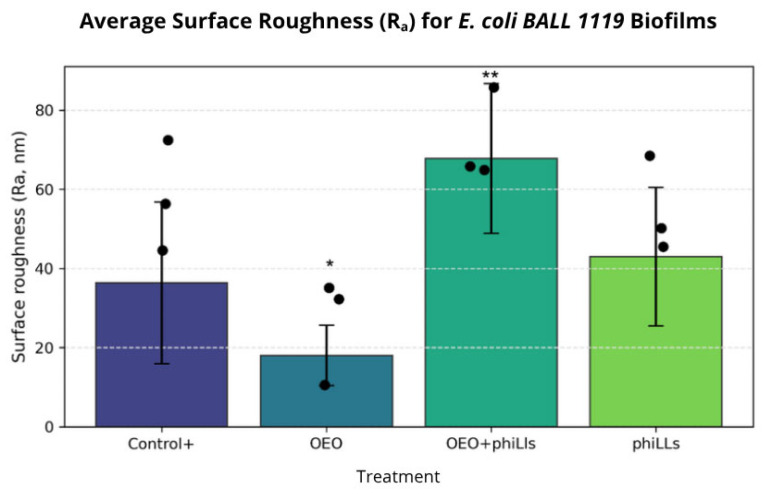
Average surface roughness (Ra, nm) ±  SD of *Escherichia coli BALL1119* biofilms after four treatments: untreated Control+ biofilm, oregano essential oil (OEO), OEO combined with phage phiLLS (OEO + phiLLS), and phage phiLLS alone. Bars represent arithmetic means of three biological replicates; black dots show individual replicate values. Error bars denote standard deviation. One-way ANOVA followed by Tukey’s multiple comparison test was used to evaluate differences among treatments. * *p* < 0.05 versus Control; ** *p* < 0.001 versus all other groups.

Notably, the combined OEO + phiLLS treatment produced the most extensive morphological disruption, characterized by deep valleys, sharp ridges, and loss of structural cohesion, suggesting synergistic degradation of the biofilm matrix. Quantitative surface roughness analysis (Figure 8) revealed statistically significant differences among treatments. The OEO + phiLLS combination significantly increased mean surface roughness (Ra) compared to the untreated control (*p* < 0.001) and to each treatment (*p* < 0.001 vs. OEO; *p* < 0.001 vs. phiLLS). These results confirm that the morphological changes observed in AFM micrographs are quantitatively measurable. The enhanced effect of the combined treatment supports a dual-mode mechanism in susceptible hosts, where OEO-mediated membrane and matrix destabilization facilitates the accessibility and action of phage-derived lytic enzymes.

## 3. Discussion

### 3.1. Combined Application of OEO and Phage

The combined application of bacteriophage phiLLS and OEO produced species-dependent effects on bacterial growth and biofilm structure. Among the six pathogens tested, only *E. coli* BALL1119 exhibited a clear synergistic interaction in terms of planktonic growth inhibition, with a Bliss synergy index of 10.8% at 9 h (Figure 2B), exceeding the accepted threshold. Growth kinetic modeling confirmed that phage phiLLS alone induced a rapid but transient inhibitory effect between 1–4 h, whereas OEO and the combination sustained inhibition over time. In contrast, all other tested strains, including *S. aureus*, *Listeria monocytogenes*, and *Salmonella*, exhibited only additive interactions with Bliss values below 10%, suggesting that the combined treatment may confer beneficial, but not fully synergistic, effects in non-host or partially resistant species.

### 3.2. Biofilm Inhibition Patterns Across Species

The combined action of oregano essential oil (OEO) and phage phiLLS in inhibiting biofilm formation varied notably among species, highlighting a complex interplay between treatment efficacy, bacterial susceptibility, and synergy interactions. In crystal violet (CV) assays, *Staphylococcus aureus* and *Salmonella* spp. biofilms exhibited the highest inhibition levels under combined treatment, with values of 71.0% and 67.8%, respectively, surpassing either agent alone (*p* < 0.05), indicating potential synergistic or additive effects (Figure 3B). In *S. aureus*, this inhibition was mirrored in the TTC assay (68.6%) and yielded a strong average inhibition (~48%), aligning well with morphological biofilm damage observed via AFM. Notably, *Salmonella* also exhibited an average TTC inhibition of nearly 44%, further supporting a biologically relevant antibiofilm effect, despite the absence of direct phage activity in planktonic growth.

However, clear synergies, as determined using both the HSA and Bliss models, were comparatively rare. As shown in Figure 4, only three out of six species, including *L. monocytogenes*, *S. aureus*, and *Salmonella*, exhibited synergy (Δ > 10%) in CV biofilm inhibition, with *S. aureus* being the only species showing synergy in both CV and TTC. *B. cereus* biofilms, conversely, showed neither inhibition nor synergy and were flagged as antagonistic under both models. Discrepancies between HSA and Bliss (e.g., *Listeria* TTC) highlight the use of a phage lytic only for *E. coli* strains.

The two *E. coli* strains exhibited mixed responses. Although *E. coli* BALL1119 demonstrated synergy in planktonic growth inhibition (Bliss 10.8%), it did not display clear synergy in biofilm inhibition, particularly under TTC, perhaps reflecting weaker phage activity within mature biofilm matrices or greater susceptibility to wash-induced detachment artifacts during assay processing.

These findings emphasize the value of combining quantitative synergy models (HSA, Bliss) with morphological and kinetic analyses to fully capture the dynamics of treatment interaction. The Bliss model revealed hidden antagonism when one agent lacked standalone efficacy, while the HSA model identified practical improvements relative to the best single agent. Such divergence highlights the need to consider biological context and matrix complexity when interpreting synergy within biofilm systems.

### 3.3. Methodological Considerations and Limitations

Methodologically, we recognize several factors that may have influenced results. In the TTC assay, which reflects metabolic activity, weak respiration or detachment of disrupted but viable cells may confound true inhibition. Likewise, washing steps in assays may unevenly affect fragile biofilms, as seen in *E. coli*, potentially diminishing treatment impact. Future experiments could benefit from gentler handling protocols, dynamic real-time monitoring, or imaging-guided biofilm quantification to reduce procedural bias. Additionally, testing across a broader OEO concentration range and multiple MOIs may better delineate synergy thresholds across species.

### 3.4. AFM-Based Insights on Biofilm Architecture Disruption

In the context of biofilm inhibition and eradication, however, the observed effects extended beyond planktonic inhibition. Quantitative assays (CV and TTC) revealed significant reductions in biomass and metabolic activity in both *E. coli* BALL1119 and *S. aureus* biofilms under the combined treatment. These findings were corroborated by AFM imaging, which showed deep structural damage to the biofilm surface in both species. While *E. coli*, a known host of phiLLS, displayed extensive disruption likely caused by viral lysis and enzyme-mediated degradation, *S. aureus*, despite being a non-host, also exhibited substantial morphological collapse. This may result from the facilitation of phage-derived enzyme activity by OEO-mediated matrix destabilization, highlighting a possible non-infective, cross-species antibiofilm mechanism.

While high biological variability in *S. aureus* biofilm architecture precluded statistically significant pairwise comparisons in roughness parameters, the AFM topographical images and consistent trends in Ra/Rz values clearly showed enhanced structural disintegration in the combined treatment group, visually corroborating the significant reductions in biomass and metabolic activity. These clear visual trends were limited, possibly due to intrinsic heterogeneity in biofilm morphology or the sensitivity of AFM roughness metrics. Furthermore, variations in biofilm resilience to mechanical disturbance during rinsing steps could have biased the CV/TTC assays, particularly in loosely structured biofilms (e.g., those of *E. coli* or *Listeria*).

Taken together, these results support a dual-layered model. In susceptible hosts like *E. coli*, phage replication combined with OEO generates a robust and synergistic effect; however, in non-hosts, OEO may unmask EPS targets for phage-derived enzymes, leading to structural degradation without infection. This highlights the potential of combining phage-derived molecules with plant-based antimicrobials as a modular strategy for biofilm control across taxonomically diverse pathogens.

### 3.5. AFM-Based Insights into Antibiofilm Activity Against S. aureus and E. coli BALL 1119

Atomic force microscopy (AFM) provides a powerful platform for nanoscale interrogation of biofilm structure and mechanics, yielding quantitative parameters such as average roughness (Ra), peak-to-valley depth (Rpv), and local stiffness, which correlate directly with antimicrobial efficacy. Foundational studies by Eaton et al. [41] used AFM to show that chitosan derivatives induce cell wall collapse and a 45% drop in Ra for *S. aureus* and *E. coli*, linking morphological disruption to reduced viability. Similarly, Perry et al. [42] demonstrated dose-dependent membrane perforations in *E. coli* and surface irregularities in *S. aureus* following treatment with aqueous garlic extract and ampicillin, with AFM-measured stiffness changes mirroring antimicrobial potency. Building on these paradigms, more recent work has applied AFM to novel antibiofilm agents. Pei et al. [43] found that apigenin-7-O-glucoside at subinhibitory doses disrupted *S. aureus* and *E. coli* biofilm matrices, producing a 60–80% reduction in exopolysaccharide-coated microcolonies and a 40% decrease in Ra. Liu et al. [44] compared tea tree and rosemary oils, observing flattened surfaces versus pitted morphologies, respectively, differences that corresponded to each oil’s penetration and lytic profiles against mature biofilms. In the realm of nanomaterials, Alavi et al. [45] used AFM to show that Fe_3_O_4_ nanoparticles fragmented EPS networks and increased Ra by 50%, while Al-Dujaily et al. [46] combined AFM and force spectroscopy to confirm that biogenic silver nanoparticles reduced biofilm thickness by 30% and local stiffness by 25%. Engineered surfaces have likewise benefited. Mahmoudi-Qashqay et al. [47] correlated a 99.9% drop in *S. aureus* viability on Ti–Cu thin films with a 70% decrease in surface roughness, underscoring AFM’s role in anti-adhesive surface design.

In the present study, AFM scans (20 µm × 20 µm) of *S. aureus* and *E. coli* BALL 1119 biofilms treated with oregano essential oil (OEO) revealed a pronounced smoothing of the EPS canopy. Average peak-to-valley depths fell below 0.8 µm (valleys rarely exceeding –0.3 µm and peaks under +0.5 µm), and Ra decreased by approximately 35% relative to controls. This flattening indicates disruption of polysaccharide networks and weakened cell–cell cohesion, akin to the 45% Ra reduction reported for chitosan treatments (Eaton et al., 2008) and the smoothing effects observed with rosemary oil [44]. Such matrix loosening likely enhances agent diffusion and primes biofilms for subsequent interventions.

Strikingly, the combined OEO + phiLLS treatment generated extreme surface fracturing. AFM maps (40 × 40 µm) exhibited Ra spiking to ≈68 nm and Rpv surpassing 1.2 µm, with biofilm layers fragmented into isolated “islands” and deep lytic pits (~–0.6 µm). This roughening reflects catastrophic EPS collapse. OEO’s membrane-disruptive action likely exposes binding sites for phiLLS, the lytic cycle of which produces localized substratum exposure and amplifies fracturing. Comparable Ra spikes have been documented for carvacrol–phage combinations against *P. aeruginosa*, where increased heterogeneity facilitated deeper antimicrobial penetration despite higher surface roughness [30].

These AFM-derived mechanical insights dovetail with our biofilm assays (CV and TTC). While OEO alone weakens matrix cohesion, the dual treatment maximizes biomass loss by coupling chemical disruption with phage-mediated lysis. Importantly, manipulating surface roughness emerges as a practical lever for biofilm control. Smoothing (via OEO) reduces cohesion and primes matrix loosening, whereas fracturing (via OEO + phiLLS) achieves catastrophic EPS failure.

As shown in Figure 8, the combined OEO + phiLLS treatment yielded the highest average surface roughness (R_a_ ≈ 68 nm) and mean height (≈300 nm) compared to either agent alone. It should be noted that no formal interaction model, such as Bliss independence [40] or Loewe additivity, was applied here to quantify synergy [48]. Nonetheless, the pronounced increase in R_a_ and mean height likely reflects extensive fragmentation of the EPS matrix, followed by re-deposition of cellular debris into sharp “peaks,” whereas deep “valleys” correspond to phiLLS-mediated lysis channels and OEO-driven polysaccharide solubilization [3].

These topographical alterations suggest a cooperative disruption of biofilm structure that parallels reports of enhanced antimicrobial action when phages are combined with small-molecule agents [44,49,50]. Liu et al. [44] demonstrated that phage–antibiotic combinations produce unique mechanistic interactions that potentiate matrix penetration, while Khong et al. [49] described solid media assays revealing improved plaque formation in the presence of subinhibitory antimicrobial concentrations. Although those studies focused on antibiotics, the underlying principle of phage-facilitated agent diffusion and debris aggregation appears applicable to our phiLLS + OEO system.

### 3.6. Mechanistic Perspectives on Synergy and EPS Targeting

Although the observed biofilm inhibition patterns, particularly in *Staphylococcus aureus*, suggest synergistic or additive effects between phiLLS and OEO, the underlying mechanisms driving these interactions remain unclear.

Given that phiLLS lacks lytic activity against *S. aureus*, the observed structural collapse may stem from non-infective, enzymatic interactions, particularly involving tail-associated depolymerases active against conserved EPS motifs such as PNAG. To explore this further, it is essential to consider both the biochemical composition of biofilm matrices and the enzymatic capabilities of phage-derived components, which may extend beyond traditional host specificity. The following section explores these factors to provide a better understanding of the cross-species antibiofilm activity observed in this study.

The evolutionary dynamics between bacteriophages and bacterial communities have driven the development of phage-encoded enzymes capable of degrading biofilm matrices, including extracellular polymeric substances (EPSs). Although traditionally considered species-specific, increasing evidence supports the broader substrate versatility of certain phage-derived depolymerases, particularly those targeting conserved structural components within biofilm EPS. Azeredo et al. [51] emphasize that phages have evolved a broad arsenal of EPS-degrading enzymes, such as endolysins, virion-associated peptidoglycan hydrolases (VAPGHs), and polysaccharide depolymerases, which act synergistically to penetrate biofilm barriers and dismantle biofilm architecture, even in multispecies contexts. These enzymes have shown activity against the EPS matrix rather than solely against bacterial cell surfaces, indicating potential cross-species functionality in degrading structural EPS shared among cohabiting organisms in polymicrobial biofilms.

Phage-encoded depolymerases, particularly tail spike proteins (TSPs), are known for their highly specific enzymatic activity against capsular polysaccharides (CPSs), exopolysaccharides (EPSs), and lipopolysaccharides (LPSs) of bacterial hosts. However, accumulating structural and biochemical evidence suggests that these enzymes might also exhibit broader substrate interaction profiles. Specifically, the review by Knecht et al. [52] indicates that some depolymerases, though evolved for host recognition, may act on conserved polysaccharide motifs shared across different bacterial taxa. This implies a potential for enzymatic activity on the EPS of non-host species, especially within polymicrobial communities where EPS components can be structurally similar or shared. These free enzymes, decoupled from direct virion adsorption, could diffuse through the biofilm matrix and exert EPS-degrading activity independently of infection. In a multispecies biofilm context, this could lead to the degradation of structural EPS produced by non-host organisms, facilitating phage dissemination or indirectly enhancing antimicrobial susceptibility in the community.

Moreover, the presence of depolymerases that target ubiquitous polysaccharide moieties such as poly-N-acetylglucosamine or mannans, common among diverse bacterial taxa, suggests that these enzymes might act on EPS produced by non-host bacteria. This is particularly relevant in mixed biofilms, where structural components are often conserved across species. As Janesomboon et al. [53] demonstrate, phage vB_AbaSI_1, although specific to *Acinetobacter baumannii* in terms of lytic infection, encodes enzymatic modules including N-acetylmuramidases and peptidases that function in EPS degradation, potentially influencing the local biofilm environment beyond the host cell lysis alone.

These observations collectively support the hypothesis that phage-derived EPS-degrading enzymes may exert activity in mixed-species biofilms, affecting non-host bacteria by disrupting the shared biofilm matrix. Such cross-species EPS degradation could potentiate antimicrobial strategies by weakening the structural integrity of polymicrobial biofilms, thereby improving the susceptibility of the entire community to immune clearance or adjunctive antimicrobials.

The possibility that depolymerases cleave structurally similar repeating units in polysaccharides of different bacterial origins supports this hypothesis. As noted, enzymatic activity is not exclusively determined by host range but also by substrate conformation and chemical composition, some of which may be conserved across genera. Hence, in natural environments where bacterial species coexist and contribute collectively to EPS architecture, phage depolymerases may play a broader ecological role than previously assumed.

The extracellular polymeric substances (EPSs) that form the matrix of bacterial biofilms differ structurally and biochemically between Gram-positive and Gram-negative bacteria. Gram-negative biofilms, such as those formed by *Pseudomonas aeruginosa*, typically contain a complex mixture of polysaccharides, proteins, extracellular DNA (eDNA), lipids, and outer membrane vesicles, with lipopolysaccharides (LPSs) playing a significant role in initial adhesion and immune evasion [54,55]. In contrast, Gram-positive biofilms, like those of *Staphylococcus aureus*, lack LPSs but are rich in teichoic acids, peptidoglycan fragments, and exopolysaccharides, with proteins and eDNA also contributing to matrix structure [55,56]. Spectroscopic analyses reveal that polysaccharide content and distribution are major sources of variance between Gram-positive and Gram-negative biofilms, with Gram-negative matrices often displaying greater heterogeneity [56]. Both types of biofilms utilize eDNA and amyloid-like proteins for structural integrity, but the specific biochemical makeup and regulatory mechanisms, such as c-di-GMP signaling, which is more prominent in gram-negatives, differ [54,55,57].

Both types share similarities in initial adhesion mechanisms, involving lipopolysaccharides in Gram-negative and cell wall glycopolymers in Gram-positive bacteria [55]. The presence of polysaccharides enhances EPS adhesion strength [58]. Despite differences, both Gram types may be susceptible to similar biofilm-disrupting strategies, such as enzymatic degradation of matrix components and inhibition of amyloid-like protein polymerization [55]. These differences influence susceptibility to antimicrobials and immune responses, with Gram-positive biofilms sometimes being more susceptible to certain treatments due to differences in EPS composition and cell wall structure [59]. Understanding these distinctions is crucial for developing targeted strategies to disrupt biofilms and treat associated infections [56,60,61,62].

In *Staphylococcus aureus*, robust biofilm formation is most strongly associated with the production of polysaccharide intercellular adhesin (PIA, also known as PNAG), teichoic acids, and extracellular DNA (eDNA), with eDNA playing a key structural role by interacting with membrane-attached lipoproteins and other positively charged proteins to stabilize the biofilm matrix [63,64,65,66,67]. *S. aureus* biofilms are further reinforced by the presence of these eDNA–protein complexes, which create an electrostatic network that enhances biofilm integrity and resistance to disruption [63,64,66]. In contrast, *Escherichia coli* biofilms are primarily structured by exopolysaccharides such as cellulose and PNAG, as well as curli fimbriae, which facilitate surface adhesion and intercellular aggregation [64,68,69]. *E. coli* biofilms also incorporate eDNA, but their matrix is uniquely influenced by the interaction of nucleoid-associated proteins (like HU), eDNA, and lipopolysaccharide (LPS), which together drive phase separation and determine the physical properties of the biofilm [64,68]. While both species utilize eDNA and polysaccharides, *S. aureus* relies more on teichoic acids and protein–eDNA interactions, whereas *E. coli* biofilms are distinguished by the presence of curli, cellulose, and LPS, with curli and cellulose being especially critical for mature biofilm architecture [64,69,70]. Thus, the most robust *S. aureus* biofilms are linked to PNAG, teichoic acids, and eDNA, while *E. coli* biofilms depend on cellulose, curli, PNAG, eDNA, and LPS for their structure and resilience [64,68,69].

Interestingly, discrepancies were observed between the outcomes of the crystal violet (CV) and TTC-based biofilm inhibition assays (as per [32] and the morphological assessments obtained via atomic force microscopy (AFM)). One plausible explanation lies in the standardized washing steps applied during the CV/TTC protocols, which may have exerted variable mechanical forces across different bacterial species. Given that the biofilms were formed by six taxonomically and structurally distinct organisms, it is likely that differential susceptibility to shear-induced detachment occurred. Biofilms with weaker matrix cohesion or lower adherence to the substrate, such as those formed by certain *E. coli* strains, may have been disproportionately disrupted during washing, leading to an underestimation of their biomass retention compared to more cohesive biofilms like those of *S. aureus* or *L. monocytogenes*.

Notably, in growth kinetics assays conducted over 9 h, where no washing steps were included, *E. coli* biofilms showed clear synergy between the bacteriophage and oregano essential oil (OEO) treatment. This suggests that the mechanical integrity of the biofilm matrix, and not necessarily the metabolic response or inhibition itself, may have confounded CV/TTC-based biomass quantification in this species.

To address this limitation in future studies, we propose the following: (i) the use of standardized, gentle-flow washing systems (e.g., microfluidic or peristaltic low-shear rinsing); (ii) implementation of real-time biomass monitoring methods such as quartz crystal microbalance (QCM) or optical coherence tomography (OCT); and (iii) parallel use of biofilm matrix-specific fluorescent dyes coupled with confocal microscopy to validate biomass retention post-treatment. These refinements would enhance the accuracy of biofilm inhibition assessment, particularly in multi-species comparative studies.

The quantitative parameters extracted from atomic force microscopy (AFM) imaging, such as surface roughness and peak height, showed a clear trend toward biofilm disintegration that was more evident in the combined treatment group (phage phiLLS + OEO). This morphological trend is supported by staining-based assays (crystal violet and TTC), where significant inhibition and apparent synergy or additive effects were observed in *S. aureus* biofilms. These complementary findings suggest that the phage–OEO combination exerts a tangible impact on the biofilm matrix, even in the absence of direct phage infectivity. The lack of statistical significance in AFM data may reflect biological variability or the inherent heterogeneity of biofilm architecture. Yet, the convergence of morphological and biochemical assays supports a genuine synergistic interaction. Further investigation into the mechanistic basis of this effect, particularly the potential activity of phage-associated enzymes on non-host EPS components, warrants deeper exploration.

Despite the fundamental biological differences between *E. coli* BALL1119 (a Gram-negative host of phage phiLLS) and *S. aureus* (a Gram-positive, non-host species), the combination of phiLLS and oregano essential oil (OEO) elicited pronounced antibiofilm effects in both organisms. Atomic force microscopy (AFM) revealed that phiLLS + OEO induced the most significant morphological alterations in the biofilm architecture of both species, albeit via potentially distinct mechanisms. In *E. coli*, the effect was synergistic and statistically significant, with a marked increase in surface roughness (Ra) and deep disruption of microcolonial structure, reflecting enhanced matrix degradation likely mediated by both phage lysis and OEO-induced permeability. In *S. aureus*, non-parametric trends and visual inspection indicated surface fragmentation and collapse in the combined treatment. This suggests that, although phiLLS does not infect *S. aureus*, its extracellular enzymes, particularly a tailspike-associated depolymerase, may act on structurally analogous residues in the EPS once OEO compromises matrix integrity. These observations were reinforced by biochemical assays (crystal violet and TTC), which confirmed additive or synergistic inhibition in both species. Together, the results highlight a potential dual-action mechanism: in *E. coli*, driven by infection-dependent lysis plus matrix destabilization; in *S. aureus*, through cooperative physical and enzymatic interference with the extracellular matrix. These findings open new perspectives for cross-species biofilm control strategies using phage-derived enzymes in combination with phytochemicals. Future work should include statistical interaction analyses (e.g., Bliss or Loewe models) and correlation studies between R_a_ and functional endpoints such as biofilm coverage or viability to validate the cooperative effect observed rigorously.

## 4. Materials and Methods

### 4.1. Bacterial Strains and Culture Conditions

Bacteria were cultured on tryptic soy agar (TSA, Difco^TM^) at 37 °C for 24 h. Single colonies were picked from a TSA plate and resuspended in 5 mL of sterile tryptic soy broth (TSB, Difco^TM^). Broth was incubated under aerobic conditions at 37 °C until reaching log phase or stationary phase 18–24 h.

### 4.2. Characterization of Oregano Essential Oil

#### Instrumentation and GC–MS Conditions

The chemical composition of the *L. graveolens* (oregano essential oil) was determined with gas chromatography–mass spectrometry (GC–MS) using an Agilent 780B gas chromatograph coupled to an ion trap mass spectrometer. Separation was achieved on a VF-5 ms capillary column (30 m × 0.25 mm i.d., 0.25 µm film thickness). The oven temperature was programmed, with helium as the carrier gas at 1.0 mL min^−1^. The essential oil was injected in split mode. Electron ionization was performed at 70 eV, and mass spectra were acquired over m/z 40–400. Component identification was carried out by matching acquired spectra against the NIST 2011b library using Agilent MS WorkStation software (version 7.0.1), employing probability-based matching criteria (R-Match score). Relative abundances (%) were calculated by normalizing individual peak areas to the total ion chromatogram area (435 371 270) without the use of correction factors.

### 4.3. Phage Propagation

The bacteriophage phiLLS used in this study is maintained in the bacteriophage collection of the Laboratory of Molecular Biology and Functional Genomics at the Center for Research in Food and Development (CIAD), Culiacán Coordination. A conventional double-layer agar method [38] was used to prepare phage stock. The phage suspension was serially diluted in SM buffer, and 100 μL of each dilution was mixed with 1000 μL of *Escherichia coli* TOP10 in the logarithmic growth phase. The mixture was incorporated into molten top agar and promptly overlaid onto TSA plates. After overnight incubation at 37 °C, plates showing moderate plaque density were selected for phage harvesting. For this, 5 mL of SM buffer was added to the plate surface, and the suspension was carefully collected. The pooled supernatants were sequentially centrifuged at 8500× *g* for 15 min, 10,000× *g* for 15 min, and finally at 40,000× *g* for 4 h at 4 °C to remove bacterial debris. The resulting supernatant was filtered through a sterile 0.25 μm cellulose acetate filter and stored at 4 °C as the phage stock until further use.

### 4.4. Determination of Phage Titer

Serial 10-fold dilutions of the phage suspension were prepared in SM buffer. From each dilution, 100 µL were mixed with 1000 µL of *E. coli* TOP 10 culture in the logarithmic growth phase, and the mixtures were processed using the standard double-layer agar method [19]. The plates were incubated at 37 °C for 18–24 h, after which individual plaques were counted. The results were expressed as plaque-forming units per milliliter (PFU/mL).

### 4.5. Phage Sensitivity to OEO

The effect of oregano essential oil (OEO) on the viability of phiLLS was assessed. OEO was obtained from ORE^®^ (Chihuahua, México) and first prepared as a stock solution at 100 mg/mL in 98% ethanol. This stock was diluted in tryptic soy broth (TSB) to obtain a working solution of 50 mg/mL, which was further adjusted to the desired final concentrations (0.5–4 mg/mL) by mixing with a freshly prepared 2% agar solution to stabilize the oil [71]. Phage suspensions were incubated with the OEO preparations at 37 °C for 24 h. Controls consisted of phage suspensions without OEO, prepared with ethanol and 2% agar in TSB. Phage viability was determined after incubation using the conventional double-layer agar method [19]. Each condition was tested in two independent experiments, with duplicate plaque assays performed for each replicate.

### 4.6. Determination of MIC and MBC Values of OEO Against Foodborne Pathogens

The minimum inhibitory concentration (MIC) and minimum bactericidal concentration (MBC) of oregano essential oil (OEO) were determined following a modified broth microdilution method described by the Clinical and Laboratory Standards Institute. The assay was performed in sterile 96-well microtiter plates. Overnight cultures of *Escherichia coli* O157:H7, *E. coli* BALL 1119, *Salmonella spp*., *Listeria monocytogenes*, *Staphylococcus aureus*, and *Bacillus cereus* were grown at 37 °C with shaking at 120 rpm until reaching the logarithmic phase. Cultures were adjusted in Mueller–Hinton broth (MHB) to an OD_600_ of 0.1 (≈1 × 10^8^ CFU/mL) and then diluted to 1 × 10^6^ CFU/mL. We initially prepared OEO as a stock solution at 100 mg/mL in 98% ethanol, diluted in MHB to obtain a 50 mg/mL working solution, and subsequently serially diluted two-fold in MHB to achieve the desired test concentrations. Each well received 50 μL of the OEO dilution and 100 μL of bacterial suspension, followed by the addition of 50 μL of fresh MHB. Plates were gently mixed and incubated at 37 °C for 18 h. Bacterial viability was assessed by adding resazurin (Sigma-Aldrich^®^, Gillingham, UK) and incubating for an additional 4 h. The MIC was recorded as the lowest concentration without a color change [72]. For MBC determination, 10 μL from each well with no visible growth were plated onto Mueller–Hinton agar (MHA) and incubated at 37 °C for 18–24 h. The MBC was defined as the lowest concentration yielding no bacterial colonies. All assays were performed in three independent biological replicates, each with duplicate technical replicates.

### 4.7. Growth Kinetics Under Individual and Combined Treatments

Bactericidal activity over time was assessed for oregano essential oil (OEO) and bacteriophage phiLLS, both individually and in combination, using a 96-well microplate format. Overnight cultures of the six target foodborne pathogens (*E. coli* O157:H7, *E. coli* BALL 1119, *Salmonella* spp., *L. monocytogenes*, *S. aureus*, and *B. cereus*) were grown at 37 °C and adjusted to an OD_600_ of 0.1 (≈1 × 10^6^ CFU/mL).

The MIC values for OEO were 1 mg/mL for all strains except *E. coli* BALL 1119, which exhibited an MIC of 2 mg/mL. MBC values were consistently 2 mg/mL across all strains. To standardize experimental conditions for comparative analysis, the OEO concentration was adjusted to 2 mg/mL (equivalent to 1× MIC for BALL 1119 and 2× MIC for the remaining strains) for all treatments in the growth kinetics assay.

In combined treatments, OEO at 2 mg/mL was mixed with phiLLS at a multiplicity of infection (MOI) of 1. Control wells included OEO alone (2 mg/mL), phiLLS alone (MOI 1), and untreated cultures. Microplates were incubated at 37 °C, and bacterial growth was monitored for 9 h by measuring OD_600_ at hourly intervals, with brief shaking before each reading to ensure cell resuspension. Each treatment was tested in three independent biological replicates, each with duplicate technical replicates.

Synergy indices condense a two-drug interaction into a single, unitless number that tells you whether the combined effect is the following:

– greater than expected (synergy) – equal to the expected arithmetic/Bliss sum (additivity) – smaller than expected (antagonism).

The Bliss index is computed as follows [40].

Bliss independence assumes the agents act through unrelated mechanisms.

If agent A alone gives fractional inhibition *f_A_* and agent B gives *f_B_* at the same time-point, the inhibition we would expect under simple independence is expressed as follows:

*E_AB_ = f_A_ + f_B_ − f_A_f_B_*.

The observed fractional inhibition of the combination is *f_AB_* *_textobs_*. Interpretation scale (widely used in antimicrobial studies) is noted as follows:

– +10% or more → biologically meaningful synergy – –10% to +10% → essentially additive – –10% or less → antagonism

Cut-offs can be tightened or loosened depending on the assay’s precision.

The Highest Single Agent (HSA) model [39] is described as follows.

The HSA model compares the observed effect of the combination with the best single agent at the same concentrations, without making assumptions about the mechanisms of action. It is calculated as follows:

$$\mathrm{HAS}\;\mathrm{score}\;=\frac{fobs}{AB}-max\left(fA,\;fB\right)\mathrm{If}$$

where $\frac{fobs}{AB}$ is the fractional inhibition observed for the combination, and fA and  fB are the fractional inhibitions for each agent individually. This is noted as follows:

– ≥ +10% greater than the highest single agent → synergy

– Within ± 10% of the highest single agent → additive

– ≤ –10% lower than the highest single agent → antagonism

The HSA model is straightforward and does not assume any specific mechanism of action, making it a useful complementary analysis to the Bliss independence model in antimicrobial synergy studies.

Both Bliss independence and Highest Single Agent (HSA) models were applied to assess two-agent interactions, as they provide complementary perspectives. Bliss independence estimates the expected combined effect assuming the agents act through unrelated mechanisms, whereas HSA compares the observed combination effect directly to that of the most active single agent at the same concentrations, without mechanistic assumptions. Using both models allowed us to detect synergy in cases where the combination exceeded mathematical expectations (Bliss) and to apply a more conservative benchmark (HSA) when one agent exhibited limited activity against a given strain. For both models, interaction scores were interpreted using widely adopted thresholds in antimicrobial synergy studies, including ≥ +10% for biologically meaningful synergy, –10% to +10% for additivity, and ≤ –10% for antagonism, consistent with the assay’s experimental precision.

### 4.8. Biofilm Eradication Assays Based on Residual Biomass and Metabolic Activity

The ability of oregano essential oil (OEO), bacteriophage phiLLS, and their combination to eradicate preformed biofilms of foodborne pathogens was evaluated using two complementary approaches: crystal violet (CV) staining to assess residual biomass and 2,3,5-triphenyltetrazolium chloride (TTC) reduction to measure metabolic activity [32]. Biofilms of *E. coli* O157:H7, *E. coli* BALL 1119, *Salmonella* spp., *L. monocytogenes*, *S. aureus*, and *B. cereus* were established in sterile 96-well plates by incubating bacterial suspensions (OD_600_ = 0.1 in TSB supplemented with 1% *w*/*v* glucose) at 37 °C for 24 h. After incubation, non-adherent cells were removed, and wells were washed twice with sterile PBS 1X, pH 7.4. Fresh TSB-glucose was added, and the following treatments were applied: OEO at 2 mg/mL, phiLLS at a multiplicity of infection (MOI) of 1, their combination, solvent control (ethanol ≤1%), and untreated control. Two identical plates were prepared for parallel analysis: (a) TTC assay (metabolic activity): After treatment application, wells received 0.05% TTC (final concentration) and were incubated at 37 °C for an additional 24 h. Wells were then washed with PBS 1X, pH 7.4, and air-dried, and the TTC incorporated into the biofilm was solubilized with methanol. Absorbance was measured at 500 nm, and results were expressed as the percentage of metabolic activity relative to the untreated control. (b) Crystal violet assay (biomass): The second plate followed the same treatment and incubation steps, except that TTC was omitted. After the 24 h incubation, wells were washed with PBS 1X, pH 7.4, and residual biomass was stained with 0.1% CV for 30 min. Wells were washed twice with PBS 1X, pH 7.4, and air-dried, and the bound dye was solubilized with 70% ethanol. Absorbance was measured at 595 nm, and results were expressed as the percentage of residual biomass relative to the untreated control. All experiments were performed in three independent biological replicates, each with duplicate technical replicates.

### 4.9. AFM Analysis of Biofilms

Biofilms of *S. aureus* and *E. coli* BALL 1119 were cultivated directly on polished glass slides (26 × 76 mm, ~1.2 mm thickness; LAUKA) following a modified 96-well plate protocol described by [32]. Overnight cultures grown in tryptic soy broth (TSB) supplemented with 0.25% (*w*/*v*) glucose were adjusted to an optical density at 600 nm (OD_600_) of 0.1. A first 200 µL aliquot of each bacterial suspension was carefully placed onto the glass slide, allowed to settle, and partially dried for 1 h at room temperature. This process was repeated 2 more times over 6 h to obtain a thick layer of biofilm. Subsequently, a 200 µL aliquot, containing either control medium or the assigned treatment, was applied, and the slide was immediately covered with a coverslip to promote uniform treatment distribution. The slides were incubated at 37 °C for 18 h. The treatments were Control+: TSB–glucose without antimicrobial; OEO (2 mg/mL) pre-dissolved at 40 mg/mL in 2% (*w*/*v*) agar, then diluted in TSB–glucose; and phiLLS + OEO: bacteriophage phiLLS (1 × 10^8^ PFU/mL) combined with OEO (2 mg/mL) and phiLLS (1 × 10^8^ PFU/mL). After incubation, coverslips were removed, and samples were completely air-dried at ambient temperature (~1 h) before atomic force microscopy (AFM) imaging. AFM was used to evaluate surface topography and quantify roughness parameters, including average roughness (R_a_) and maximum height variation (Rz), to assess structural alterations in the biofilm matrix after treatment.

### 4.10. Statistical Analysis

Data were analyzed using Python 3.15, Minitab^®^ 21.1.0, and Julius AI^®^ (premium version, https://julius.ai/) for both parametric and non-parametric tests. Normality and variance homogeneity were assessed before choosing the statistical approach. Parametric data were evaluated with one-way ANOVA followed by Tukey’s HSD, while non-parametric data were analyzed using Kruskal–Wallis with Dunn’s post-hoc test. In the AFM assay for *S. aureus*, non-parametric analysis was used due to the lack of normality, whereas *E. coli* BALL 1119 met parametric assumptions. For synergy evaluation, both Bliss independence and Highest Single Agent (HSA) models were applied, with Δ values classified as synergistic (>10%), antagonistic (<–10%), or additive (–10% to 10%). Statistical significance was set at *p* < 0.05, and results are expressed as mean ± SD from independent experiments.

## Figures and Tables

**Table 1:** GC–MS chemical composition of *Lippia graveolens* essential oil.

Compound	Chemical Class	RT (min)	R-Match	% Composition
α-Pinene	Monoterpene hydrocarbons	3.906	849	0.870
β-Myrcene	Monoterpene hydrocarbons	4.196	841	1.750
p-Cymene	Monoterpene hydrocarbons	4.364	816	28.616
γ-Terpinene	Monoterpene hydrocarbons	4.589	798	2.885
Terpinen-4-ol	Oxygenated monoterpenes	5.657	717	0.739
Methylthymol	Phenolic derivatives/esters	5.926	910	0.295
Isothymol methyl ester	Phenolic derivatives/esters	6.006	915	0.198
Thymol	Phenolic monoterpenes	6.865	750	0.174
Carvacrol	Phenolic monoterpenes	6.520	750	58.897
Caryophyllene	Sesquiterpene hydrocarbons	7.350	783	2.593
Humulene	Sesquiterpene hydrocarbons	7.615	700	2.065
Aromadendrene	Sesquiterpene hydrocarbons	7.831	846	0.323
Caryophyllene oxide	Oxygenated sesquiterpenes	8.702	843	0.597

All identifications were tentative and based on mass-spectral matching to the NIST 2011b library using Agilent MS WorkStation software (v. 7.0.1). Relative amounts were determined by peak-area normalization without correction factors. Most of the identified compounds were monoterpenes (93.3%), dominated by phenolic monoterpenes such as carvacrol (58.90%) and p-cymene (28.62%), followed by γ-terpinene (2.89%). Sesquiterpenes accounted for 5.3% of the composition, primarily caryophyllene (2.59%) and humulene (2.07%), while oxygenated compounds such as terpinen-4-ol (0.73%) were present in minor amounts (<1%).

**Table 2:** Minimum inhibitory concentration (MIC) and minimum bactericidal concentration (MBC) of oregano essential oil (OEO) against six bacterial strains. OEO was tested at concentrations from 4.0 to 0.025 mg/mL. Symbols: (–) indicates no bacterial growth; (+) indicates bacterial growth.

48 h	OEO 2 mg/mL	OEO 1 mg/mL	OEO 0.5 mg/mL	OEO 0.25 mg/mL
*E. coli BALL* 1119	−	+	+	+
*L. monocytogenes*	−	−	+	+
*Salmonella* spp.	−	−	+	+
*S. aureus*	−	−	+	+
*E. coli* O157:H7	−	−	+	+
*B. cereus*	−	−	+	+

## Data Availability

The raw data supporting the conclusions of this article will be made available by the authors on request.

## Abbreviations

The following abbreviations are used in this manuscript:

OEO	Oregano essential oil
phage	Bacteriophage
AFM	Atomic force microscopy
CV	Crystal violet
TTC	2,3,5-Triphenyltetrazolium chloride
EPS	Extracellular polymeric substance
MIC	Minimum inhibitory concentration
MBC	Minimum bactericidal concentration
MOI	Multiplicity of infection
Ra	Average surface roughness
Rz	Maximum height variation
ΣFIC	Sum of fractional inhibitory concentration
Bliss	Bliss independence model (ΔBliss)
HSA	Highest single agent model (ΔHSA)
PFU	Plaque-forming unit
CFU	Colony-forming unit
PBS	Phosphate-buffered saline
TSB	Tryptic soy broth
TSA	Tryptic soy agar
MHB	Mueller–Hinton broth
MHA	Mueller–Hinton agar
PCA	Principal component analysis
OD6	Optical density
